# Supplementary material for: A Hybrid of Amodiaquine and Primaquine Linked by Gold(I) Is a Multistage Antimalarial Agent Targeting Heme Detoxification and Thiol Redox Homeostasis
Source: Pharmaceutics. 2022 Jun 12;14(6):1251. doi: 10.3390/pharmaceutics14061251 (PMC9229949; doi:10.3390/pharmaceutics14061251)
Supplement: Supplementary file 1 [file pharmaceutics-14-01251-s001.zip › pharmaceutics-1734642-supplementary.pdf]

pharmaceutics

Supplementary Materials: A Hybrid of Amodiaquine and Primaquine Linked by Gold(I) Is a Multistage Antimalarial Agent Targeting Heme Detoxification and Thiol Redox Homeostasis

Caroline de Souza Pereira, Helenita Costa Quadros, Samuel Yaw Aboagye, Diana Fontinha, Sarah D'Alessandro, Margaret Elizabeth Byrne, Mathieu Gendrot, Isabelle Fonta, Joel Mosnier, Diogo Rodrigo M. Moreira, Nicoletta Basilico, David L. Williams, Miguel Prudêncio, Bruno Pradines and Maribel Navarro

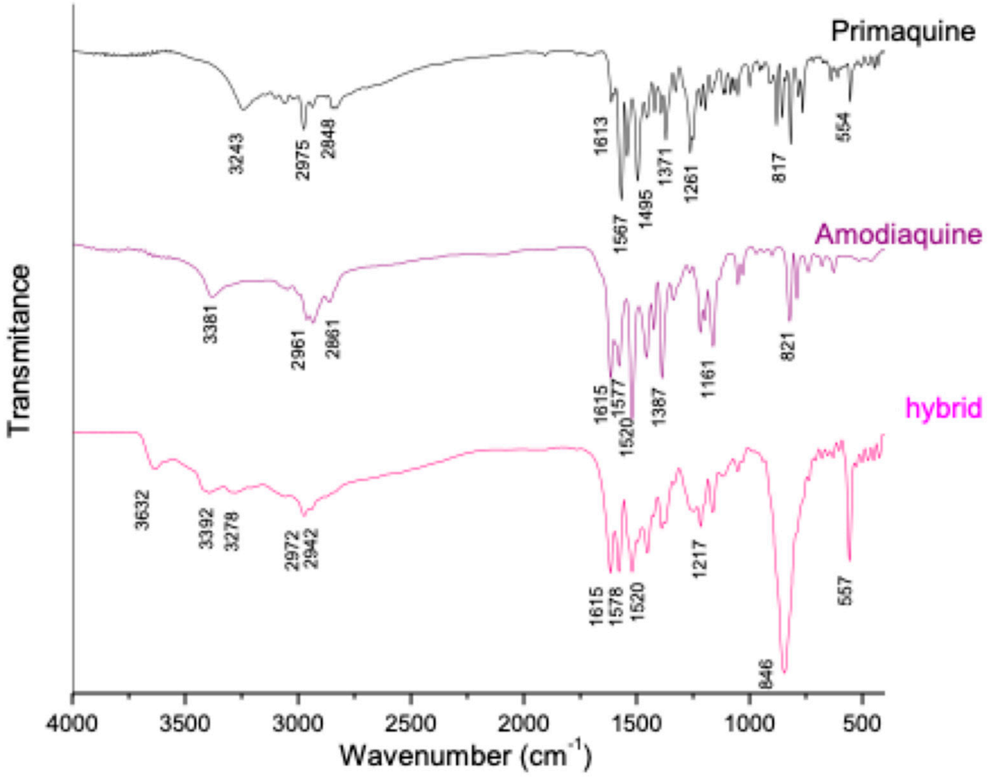

Figure S1. Infrared spectra for the quinolinic ligands and the $[AuAQPQ]PF_6$ hybrid.

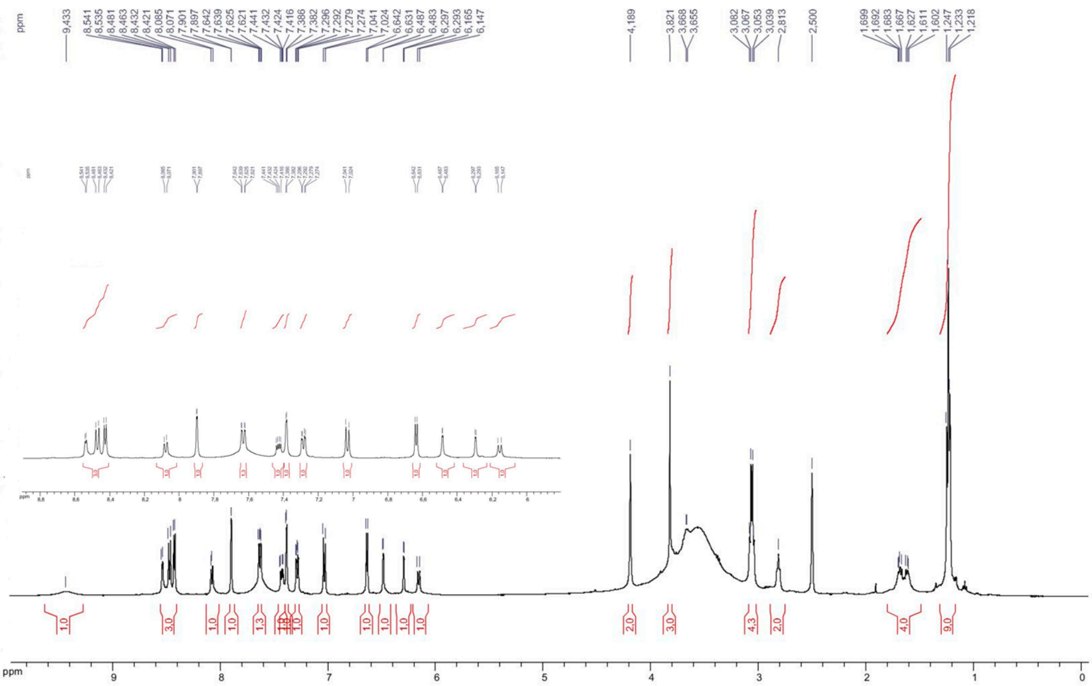

Figure S2. ^{1}H NMR (DMSO-d_6, 298K, 500 MHz) of [AuAQPQ]PF_6 hybrid.

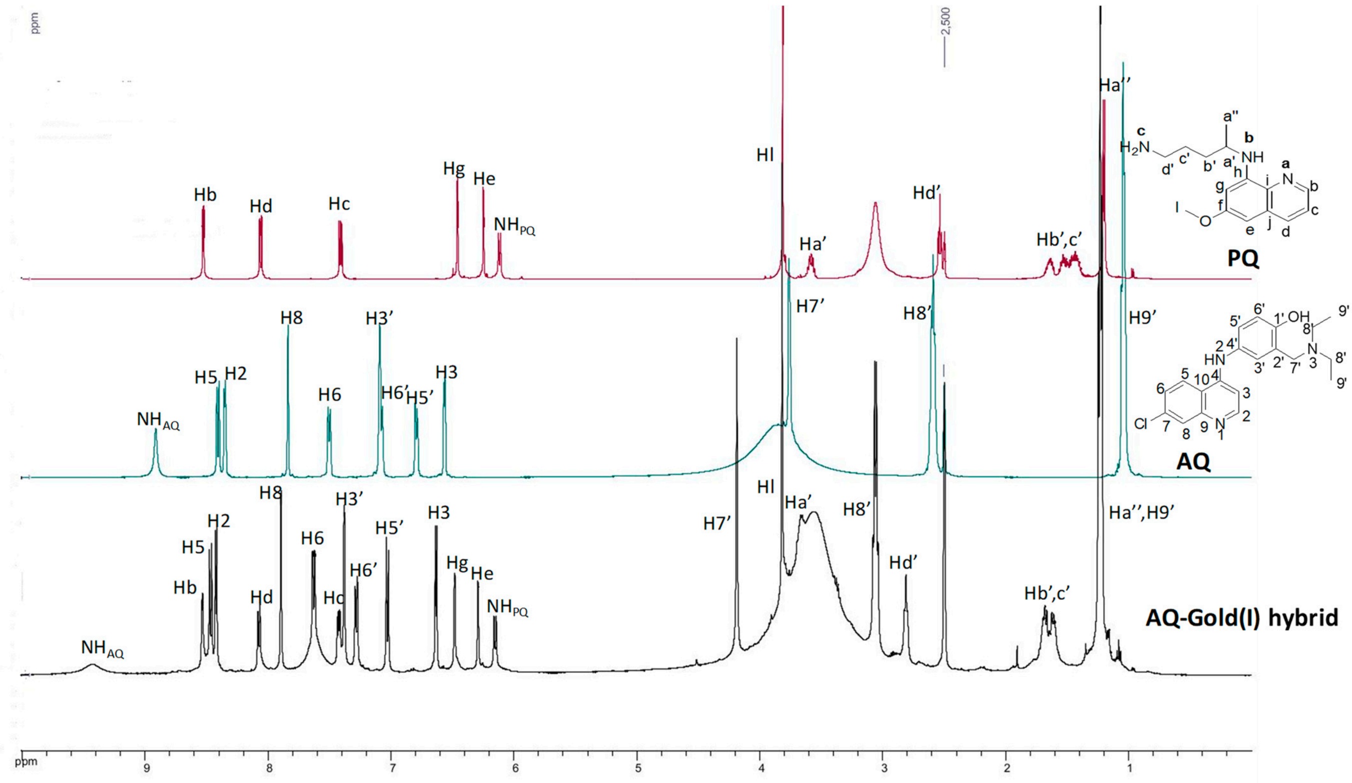

Figure S3. ^{1}H NMR (DMSO-d_6, 298K, 500 MHz) of the metal-free quinolines and of [AuAQPQ]PF_6 hybrid.

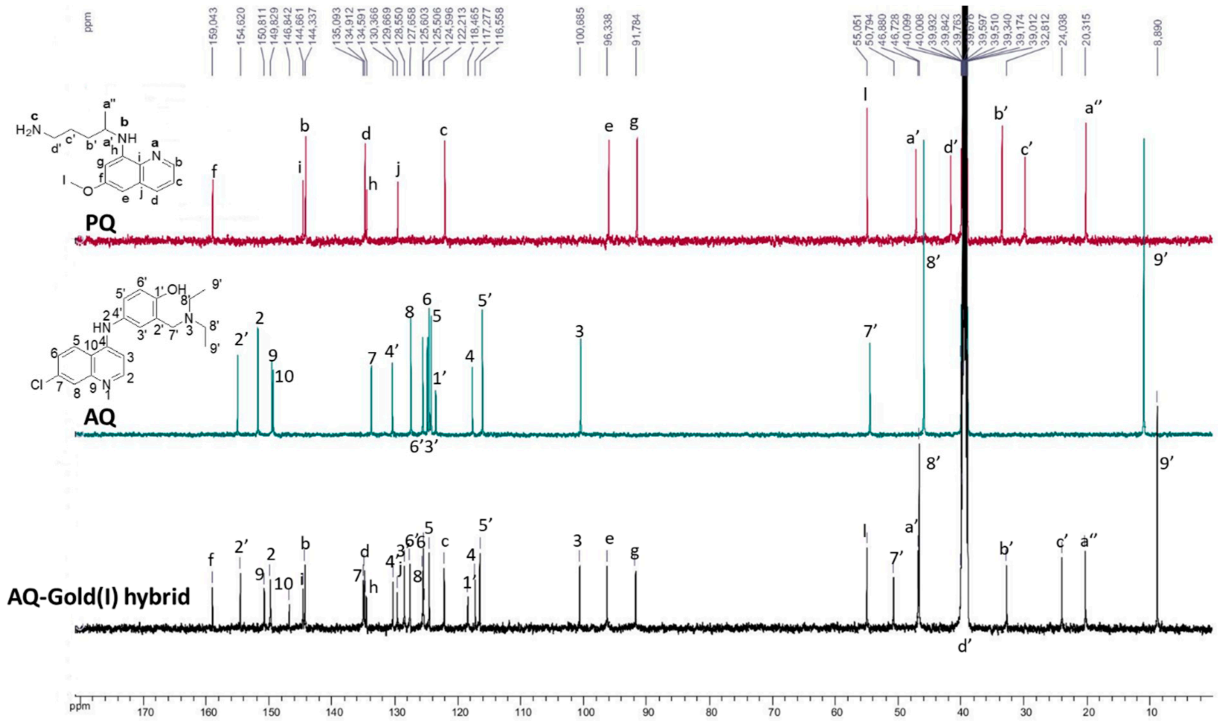

Figure S4. ^{13}C NMR (DMSO-d_6, 298K, 125 MHz) of the quinolinic ligands and of [AuAQPQ]PF_6 hybrid.

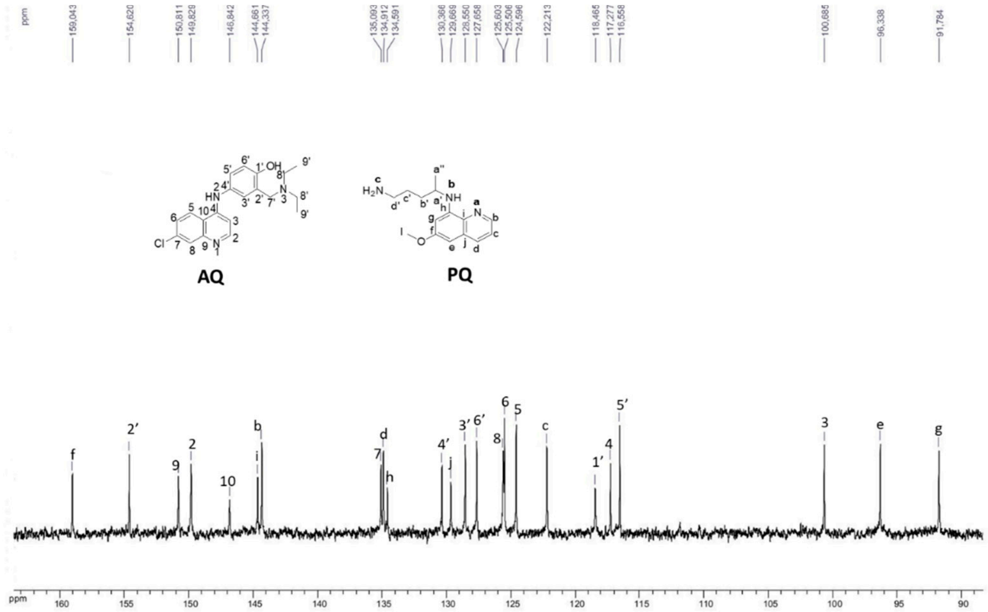

Figure S5. Expansion of ^{13}C NMR (DMSO-d_6, 298K, 125 MHz) in the aromatic region for the [AuAQPQ]PF_6 hybrid.

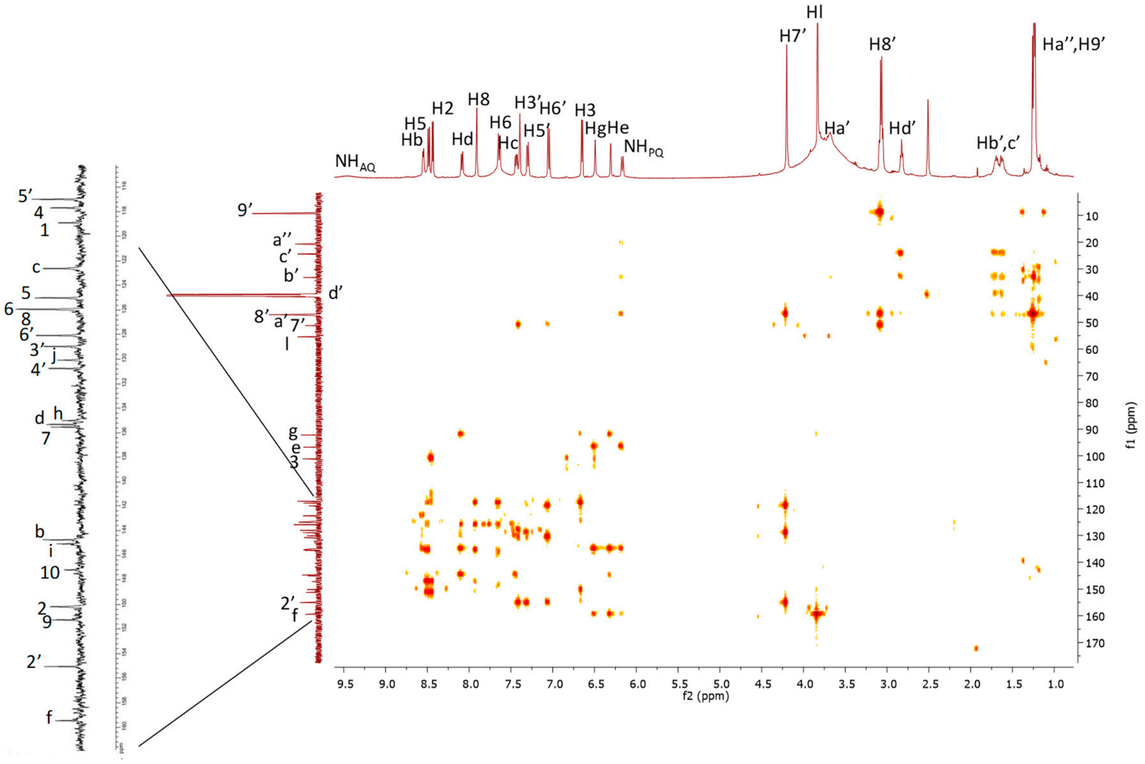

Figure S6. ^{13}C-^{1}H HMBC (DMSO-d$_6$, 298K) of the [AuAQPQ]PF_6 hybrid.

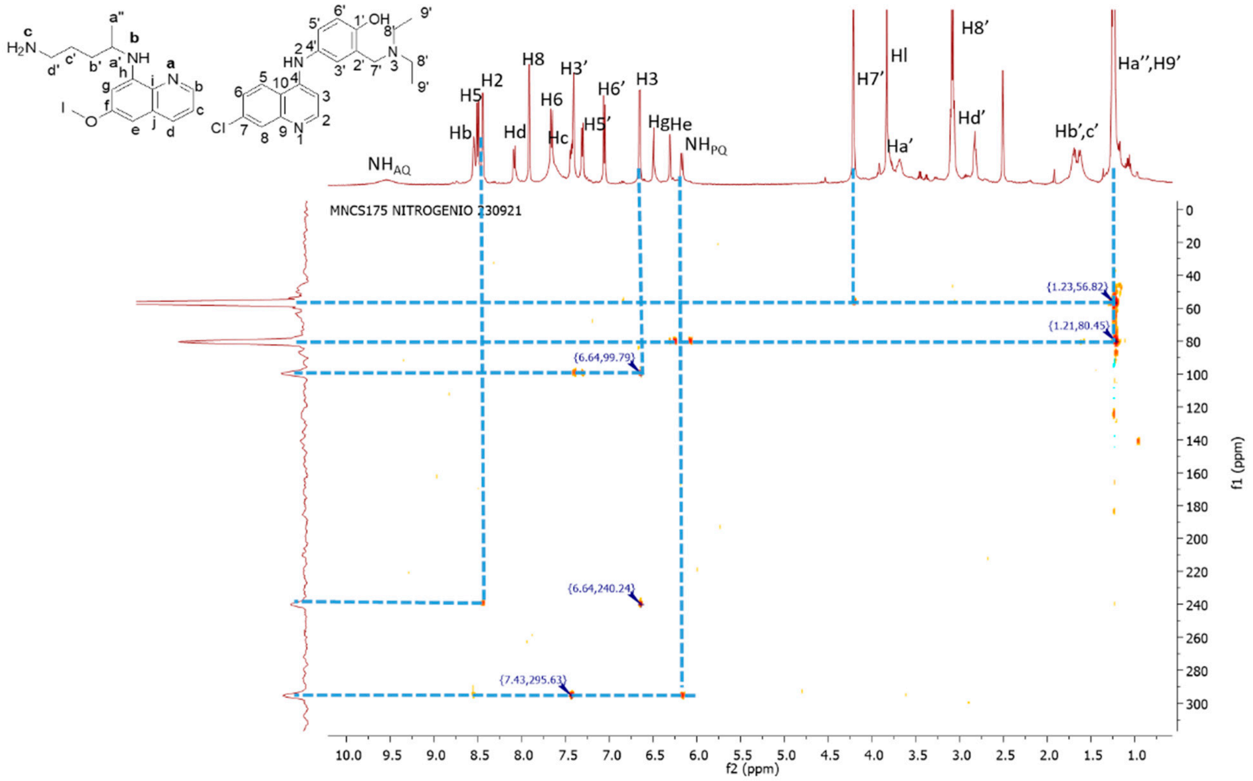

Figure S7. ^{15}N-^{1}H HMBC (DMSO-d$_6$, 298K) of the [AuAQPQ]PF_6 hybrid.

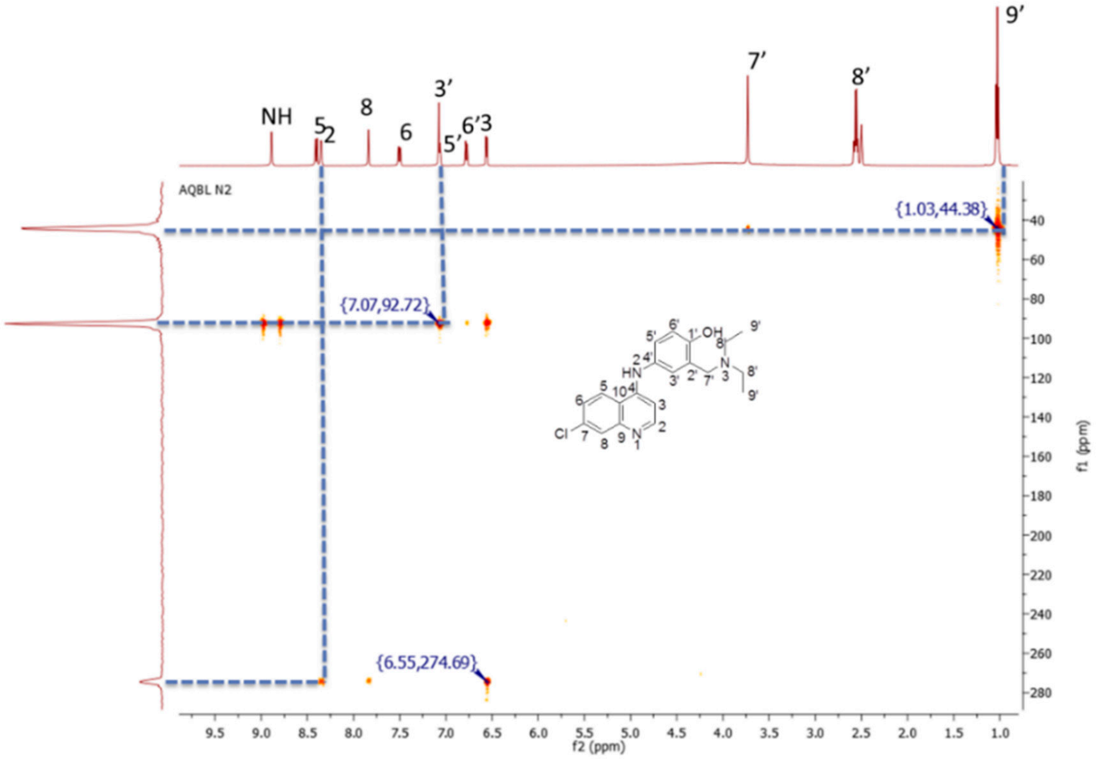

Figure S8. ^{15}N-^{1}H HMBC (DMSO-d_6, 298K) of amodiaquine (AQ).

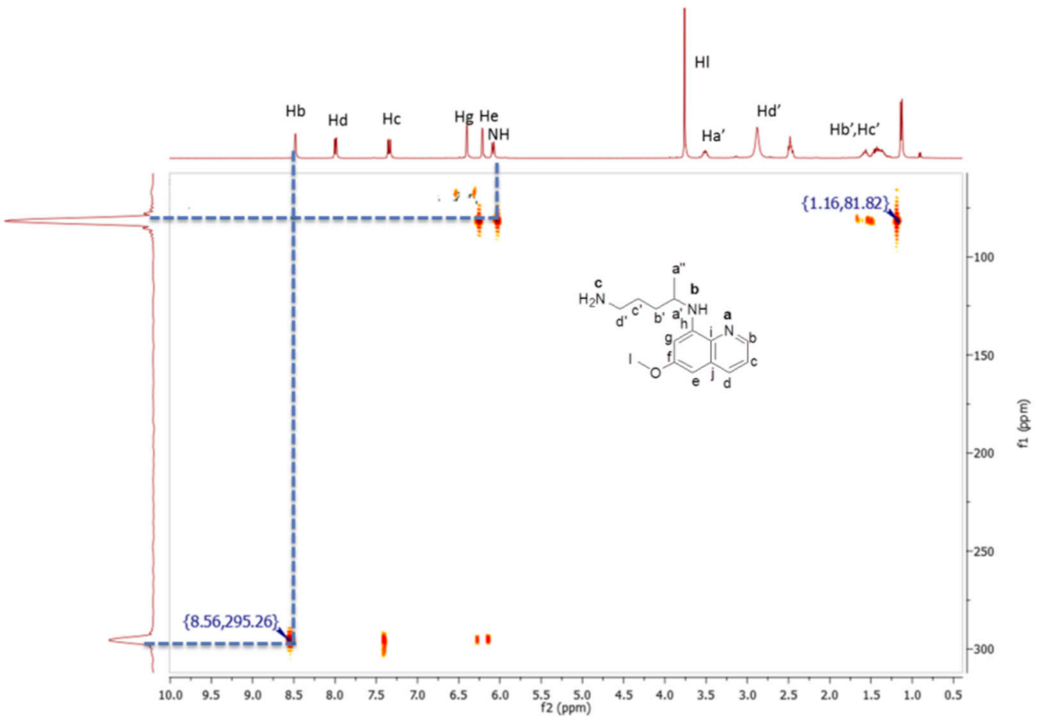

Figure S9. ^{15}N-^{1}H HMBC (DMSO-d_6, 298K) of primaquine (PQ).

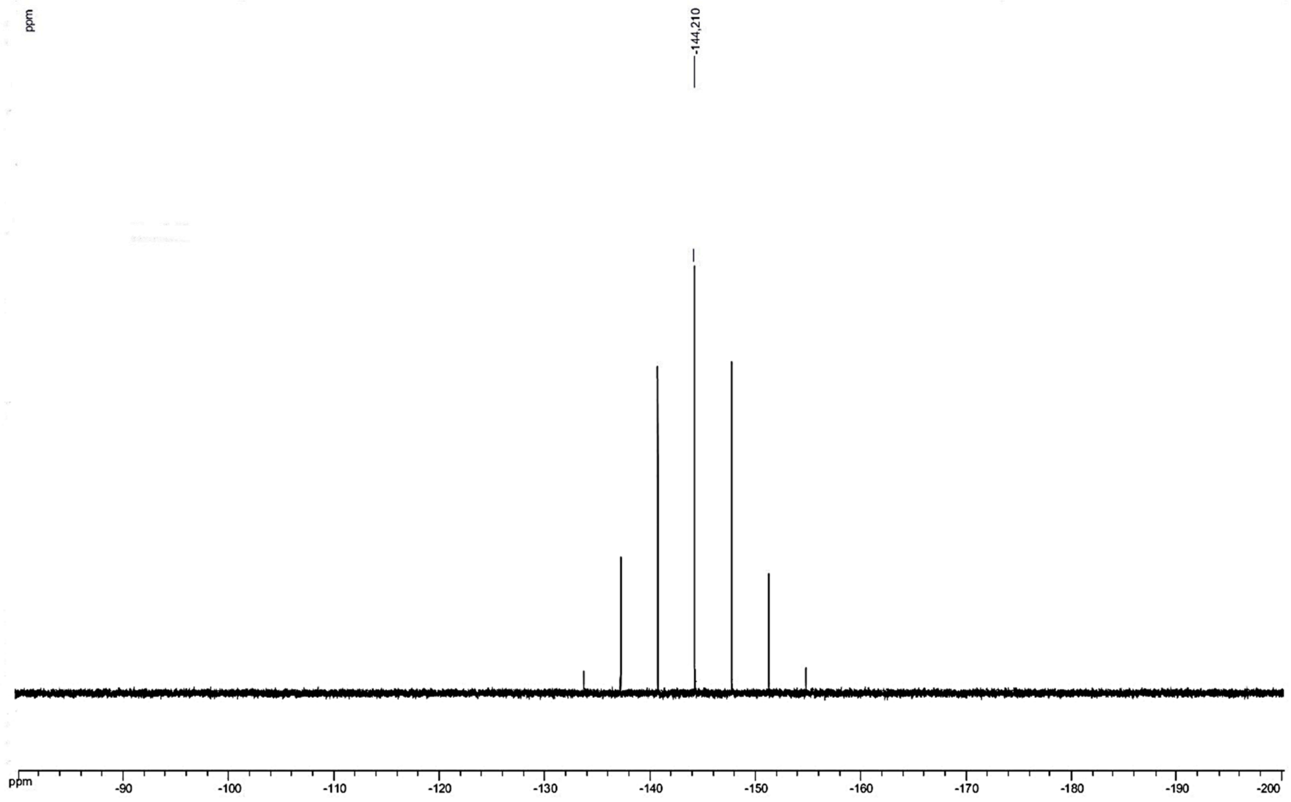

Figure S10. $^{31}P\{^{1}H\}$ NMR (DMSO-d_6, 298K, 202 MHz) of the $[AuAQPQ]PF_6$ hybrid.

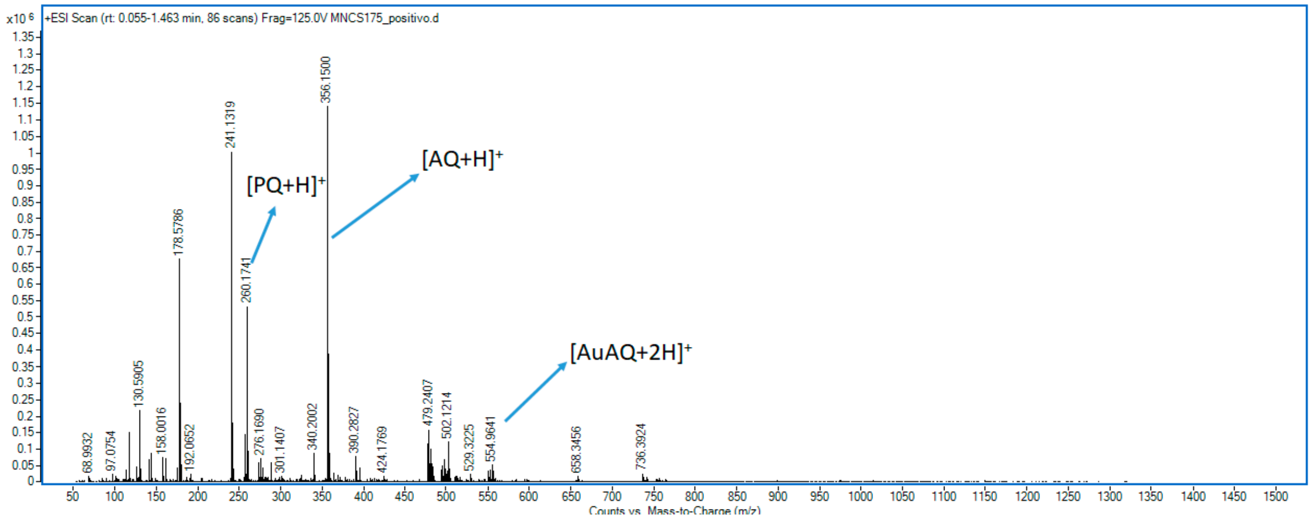

Figure S11. ESI-MS spectrum of the $[AuAQPQ]PF_6$ hybrid.

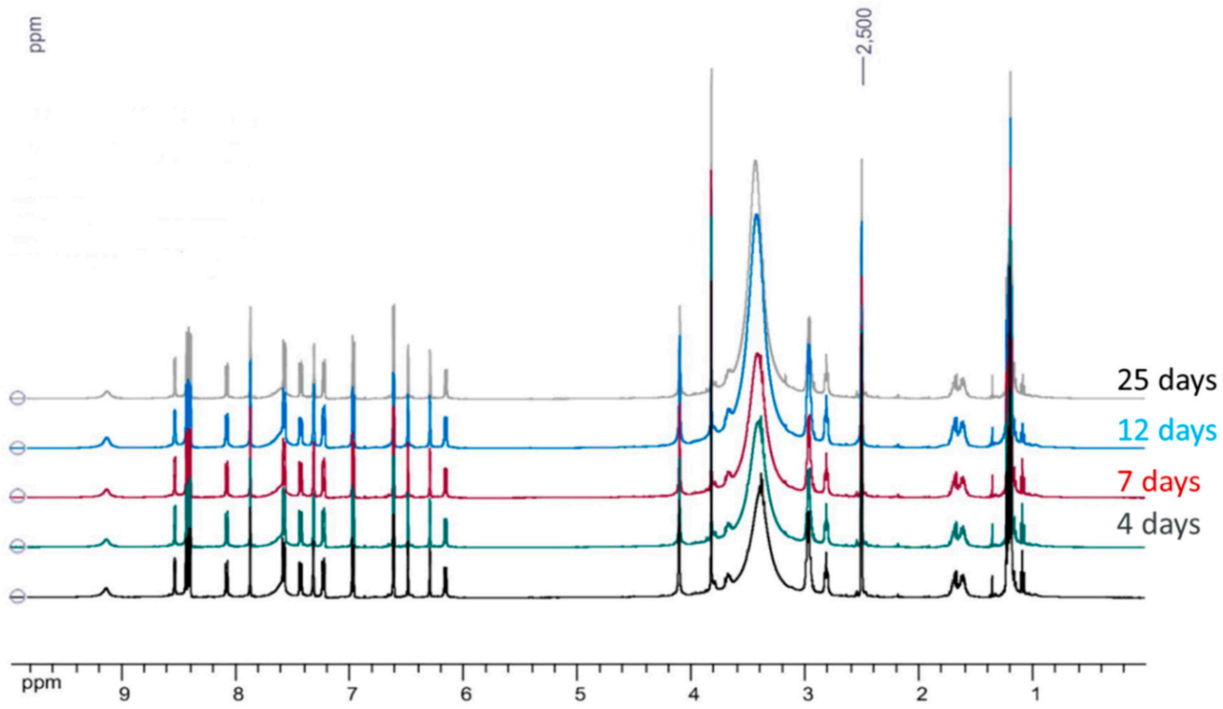

Figure S12. Stability of [AuAQPQ]PF_6 hybrid in a DMSO-d_6 solution at 298K monitored by ^{1}H NMR (500 MHz).

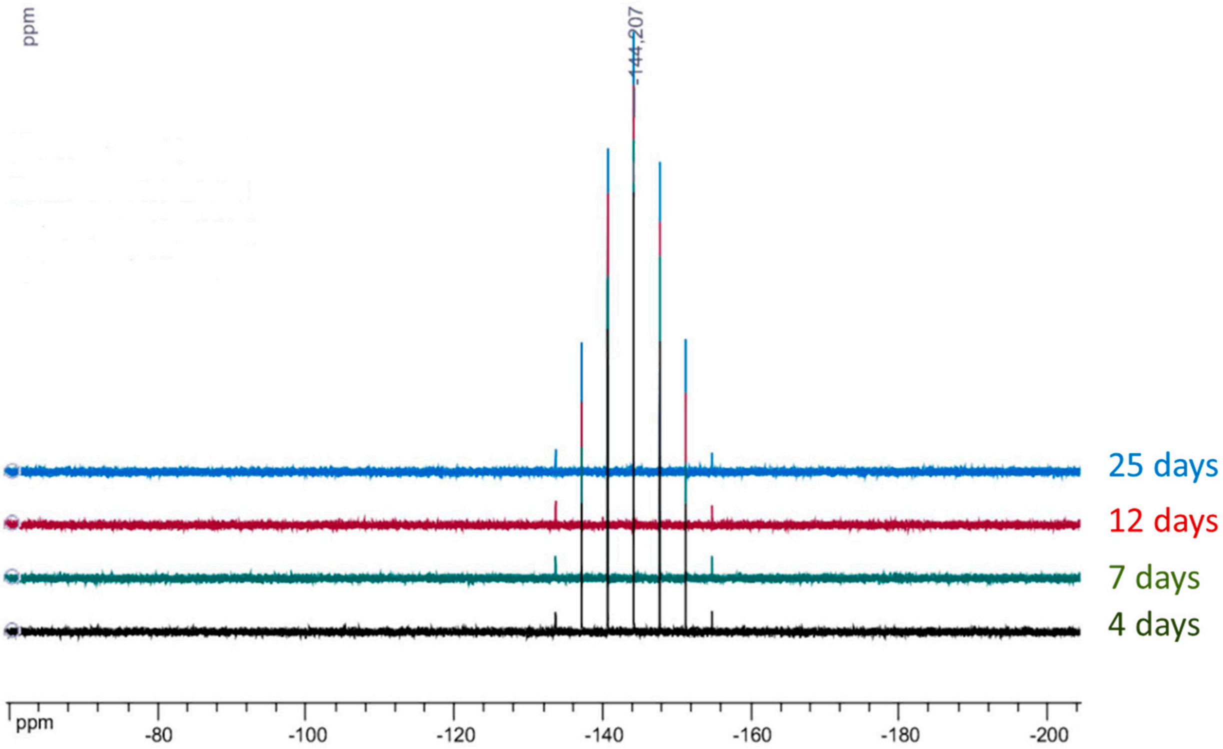

Figure S13. Evaluation of stability of [AuAQPQ]PF_6 hybrid in a DMSO solution at 298K monitored by $^{31}P\{^1H\}$ (202 MHz).

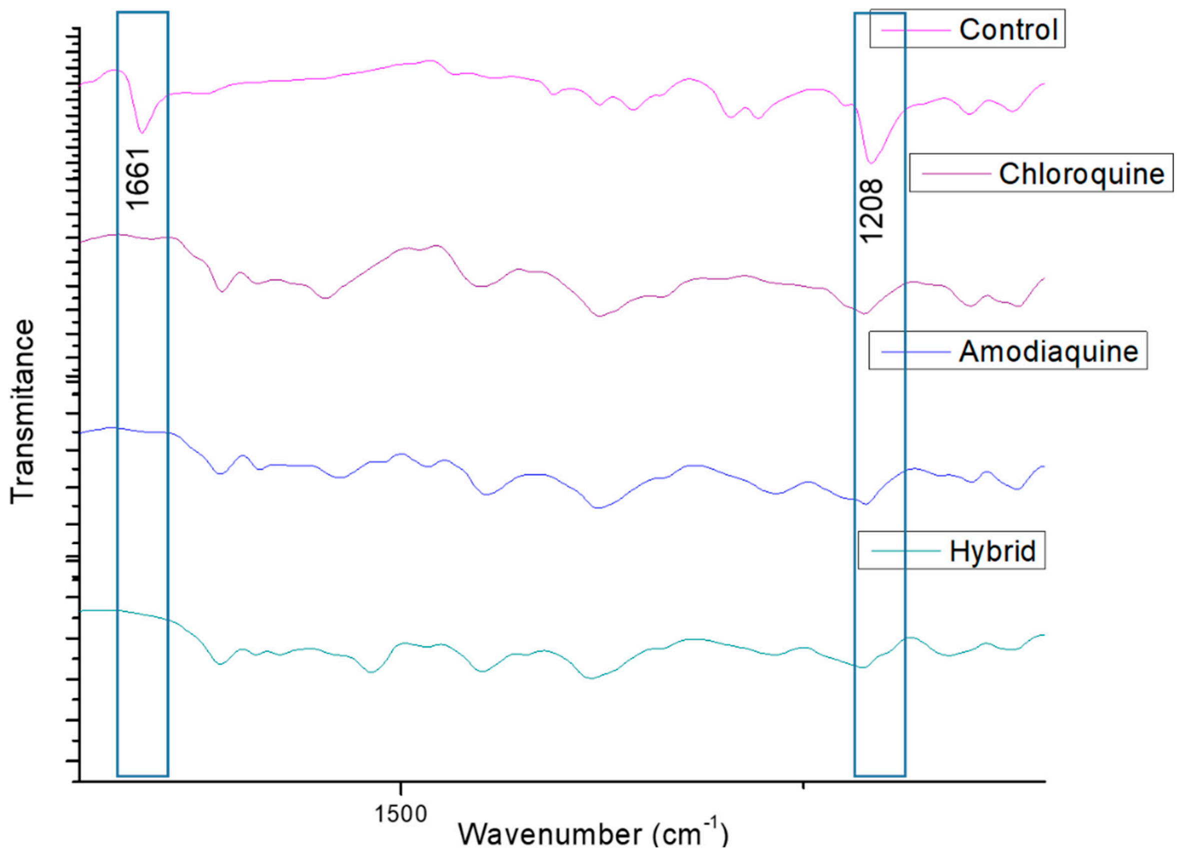

Figure S14. Formation of β-hematin monitored by IR and its inhibition by $[AuAQPQ]PF_6$ hybrid (**2**).

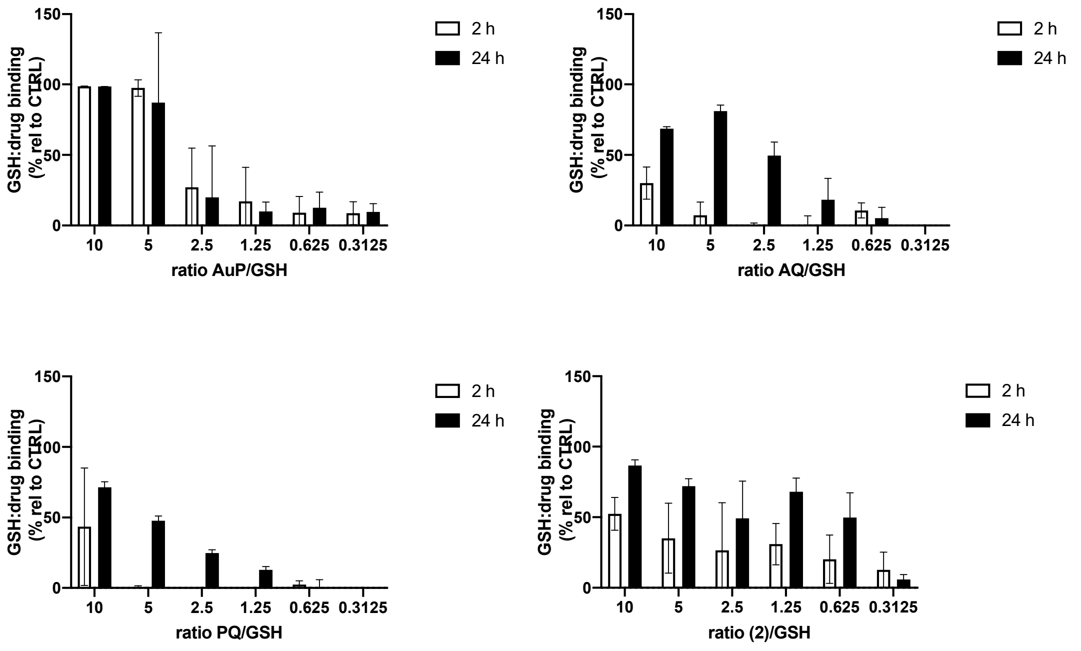

Figure S15. Interaction of compounds with reduced glutathione (GSH) at indicated time of incubation. Values are the mean ± S.D. from two independent experiments. GSH was detected using monochlorobimane and the percent of drug:GSH binding was determined in comparison to untreated samples (no drug). AuP = $[AuClPPh_3]$.

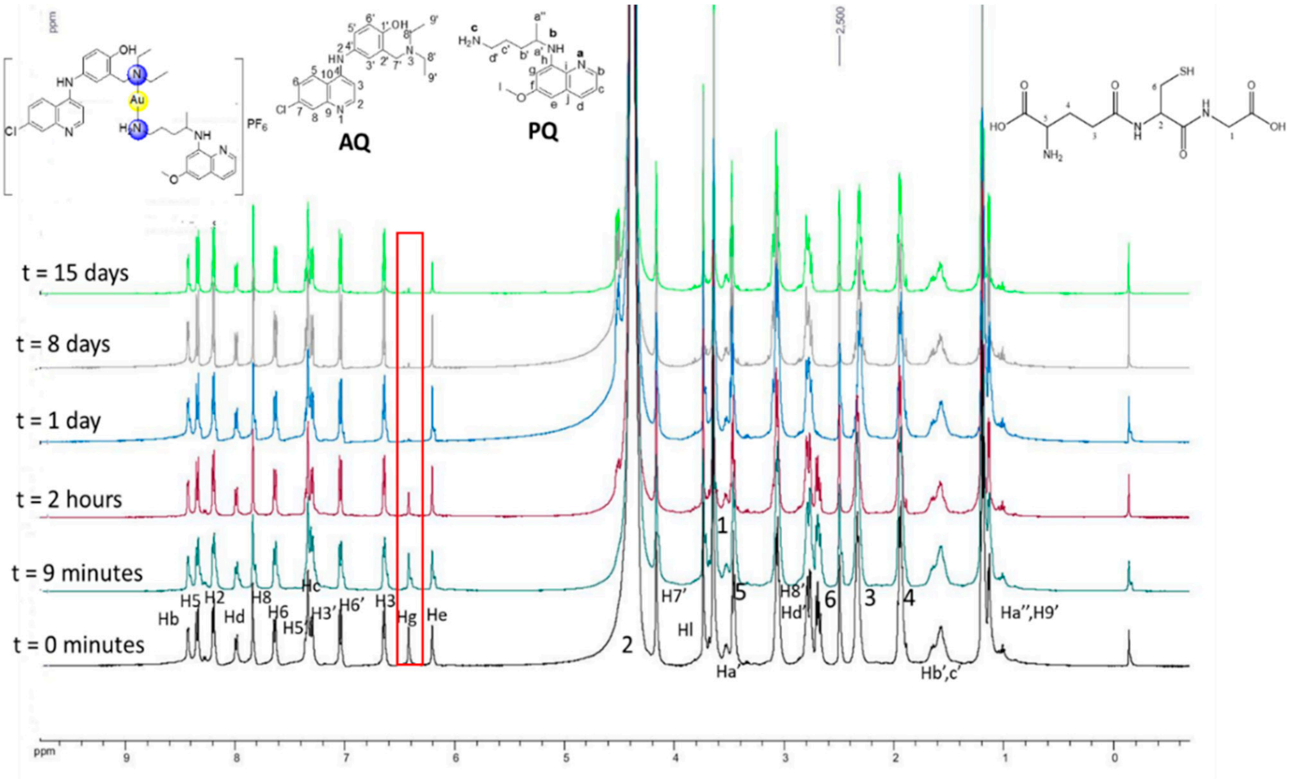

Figure S16. Evaluation of the interaction of the [AuAQPQ]PF_6 hybrid and glutathione by H^1-NMR (DMSO-d_6:D_2O / 300:200 μL, 298K, 500 MHz).
